# Supplementary material for: Understanding how excess lead iodide precursor improves halide perovskite solar cell performance
Source: Nat Commun. 2018 Aug 17;9:3301. doi: 10.1038/s41467-018-05583-w (PMC6098034; doi:10.1038/s41467-018-05583-w)
Supplement: Supplementary file 1 — Supplementary Information [file 41467_2018_5583_MOESM1_ESM.pdf]

Supplementary information for

Understanding how excess lead iodide precursor improves halide perovskite solar cell performance

Byung-wook Park et. al.

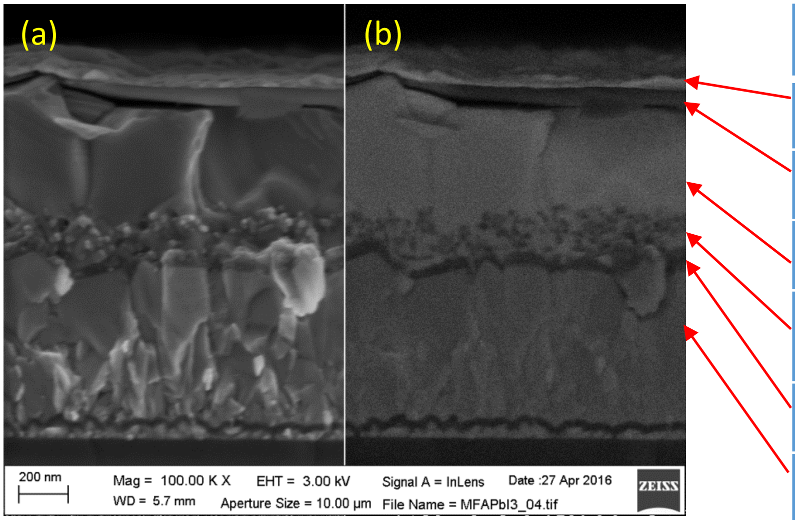

Film type	Thickness (nm)
Au	~ 80
PTAA	20 - 50
HaP	350 - 650
mp-TiO2	80 – 200
d-TiO2	40 – 60
FTO	600 - 660

Supplementary Figure 1 – Side-by-side comparison of morphology (a) and atomic number (b) contrast of the device cross section. The characteristic thickness of the various layers was not affected by the presence of excess of PbI_2 in the deposition solution.

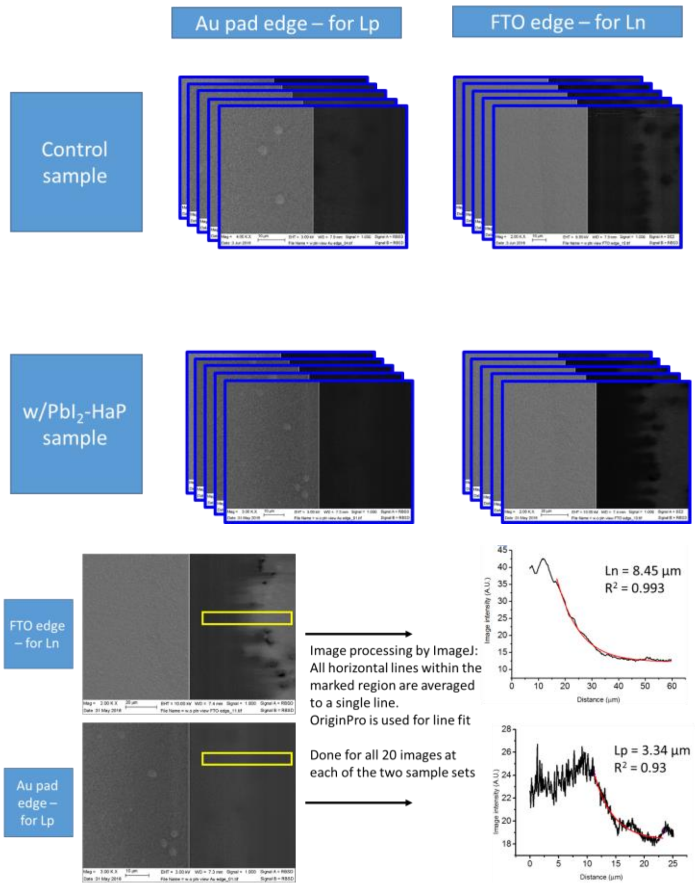

Results with fitting quality

	Lp	R^2	Ln	R^2
	2.64	0.94	7.01	0.993
	3.75	0.98	7.54	0.989
Control sample	2.86	0.95	7.63	0.989
	4.2	0.96	7.29	0.995
	2.75	0.97	7.37	0.990
	4.47	0.97	7.62	0.991
Average	3.4		7.4	
Standard deviation	0.8		0.2	
	3.34	0.93	7.42	0.992
	4.01	0.94	7.4	0.987
w/PbI_2-HaP	4.57	0.97	7.42	0.993
	3.24	0.98	7.48	0.995
	3.15	0.97	8.45	0.993
Average	3.7		7.6	
Standard deviation	0.6		0.5	

Supplementary Figure 2 – Illustration of the fitting process to extract diffusion lengths.

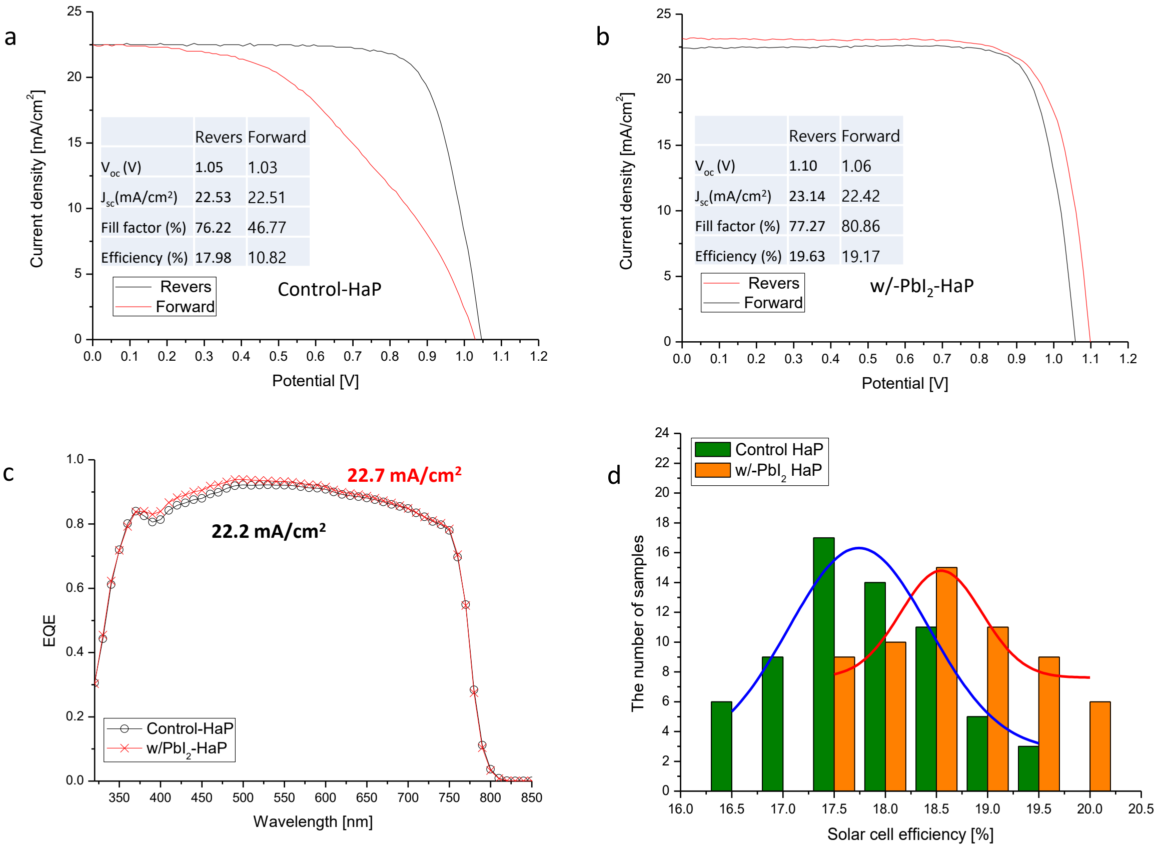

Supplementary Figure 3. J-V curves for two representative HaP solar cells: (a) high hysteresis of J-V curves for control-HaP, (b) low hysteresis of J-V curves for w/-PbI_2-HaP, (c) External quantum efficiency for cells made with control- and w/-PbI_2-HaP, and (d) Histogram of efficiencies for the cells fabricated with and without excess PbI_2. Details on the PV cell fabrication are given in the main text.

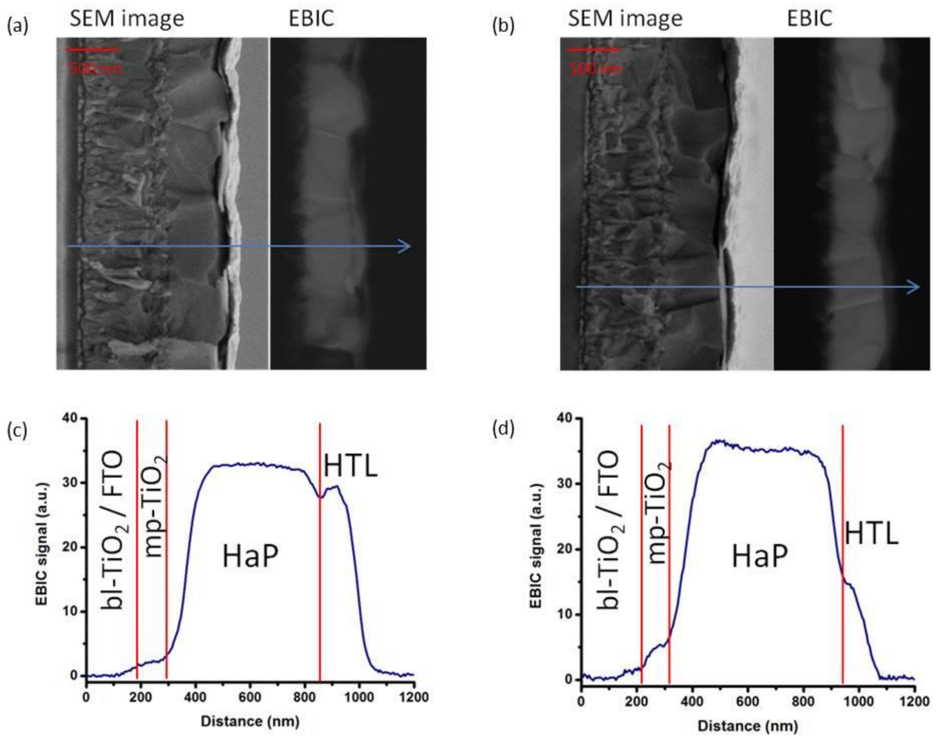

Supplementary Figure 4 – Side by side SE and EBIC images of cross-sections of cells with (a) Control-HaP and (b) w/PbI_2-HaP. The paths of the line profiles are marked with thin blue horizontal lines with arrows. The EBIC signal distributions are summarized in (c) and (d), for the two samples, using the results from the (a) and (b) images, respectively.

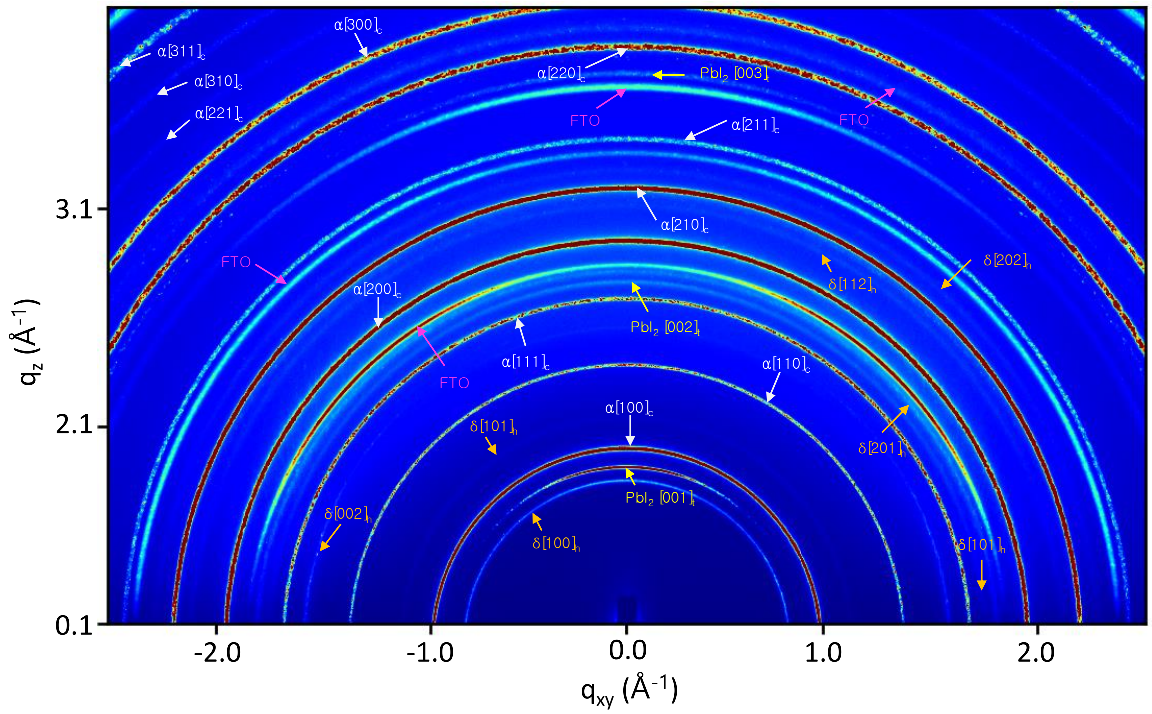

Supplementary Figure 5. Miller indexes on 2D GIWAXS pattern for FA cation-substituted HaP film on FTO.

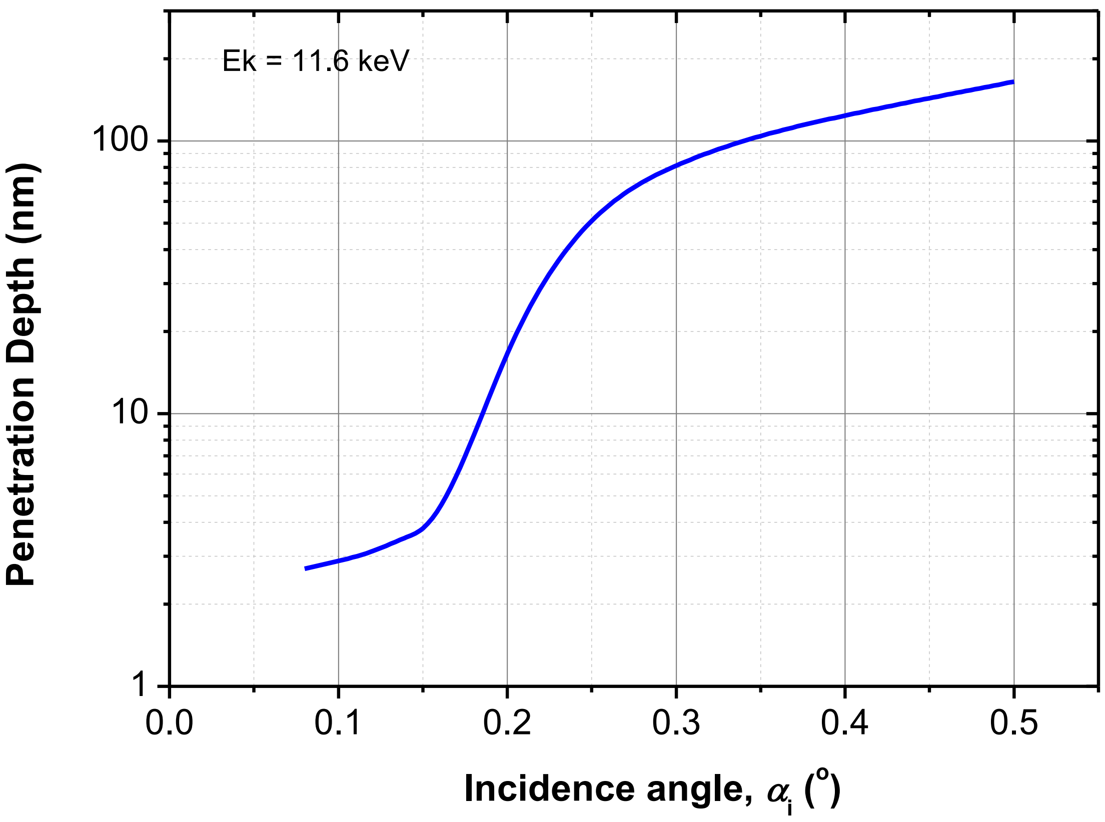

Supplementary Figure 6. X-ray penetration depth to α-$FAPbI_3$ film with x-ray incidence angles which were reported previously.[1]

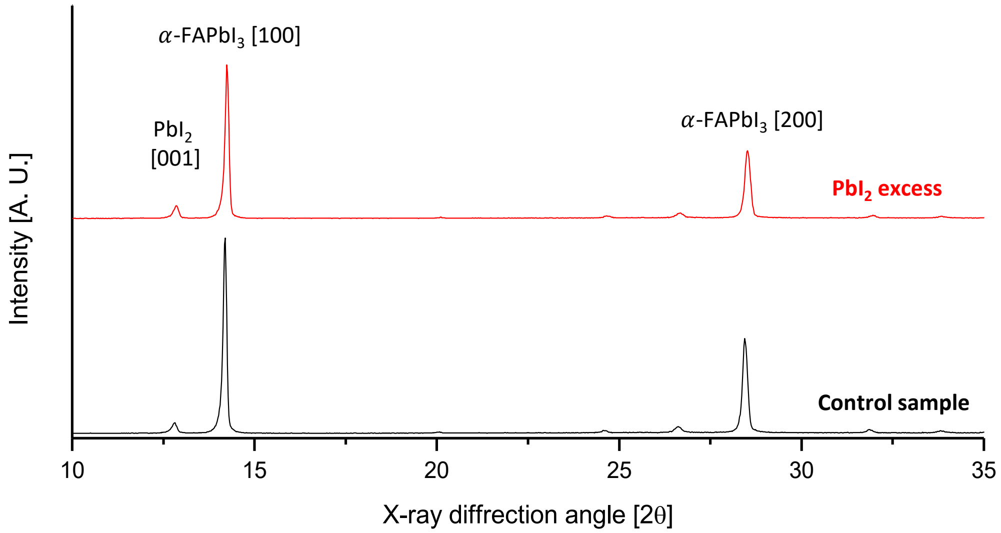

Supplementary Figure 7. Full (2θ range) XRD spectra for control-HaP and w/-PbI_2-HaP films on FTO. (XRD measurements were done on a Rigaku D/MAX2500V/PC X-ray diffractometer at 40 kV, 200 mA with a Cu target.)

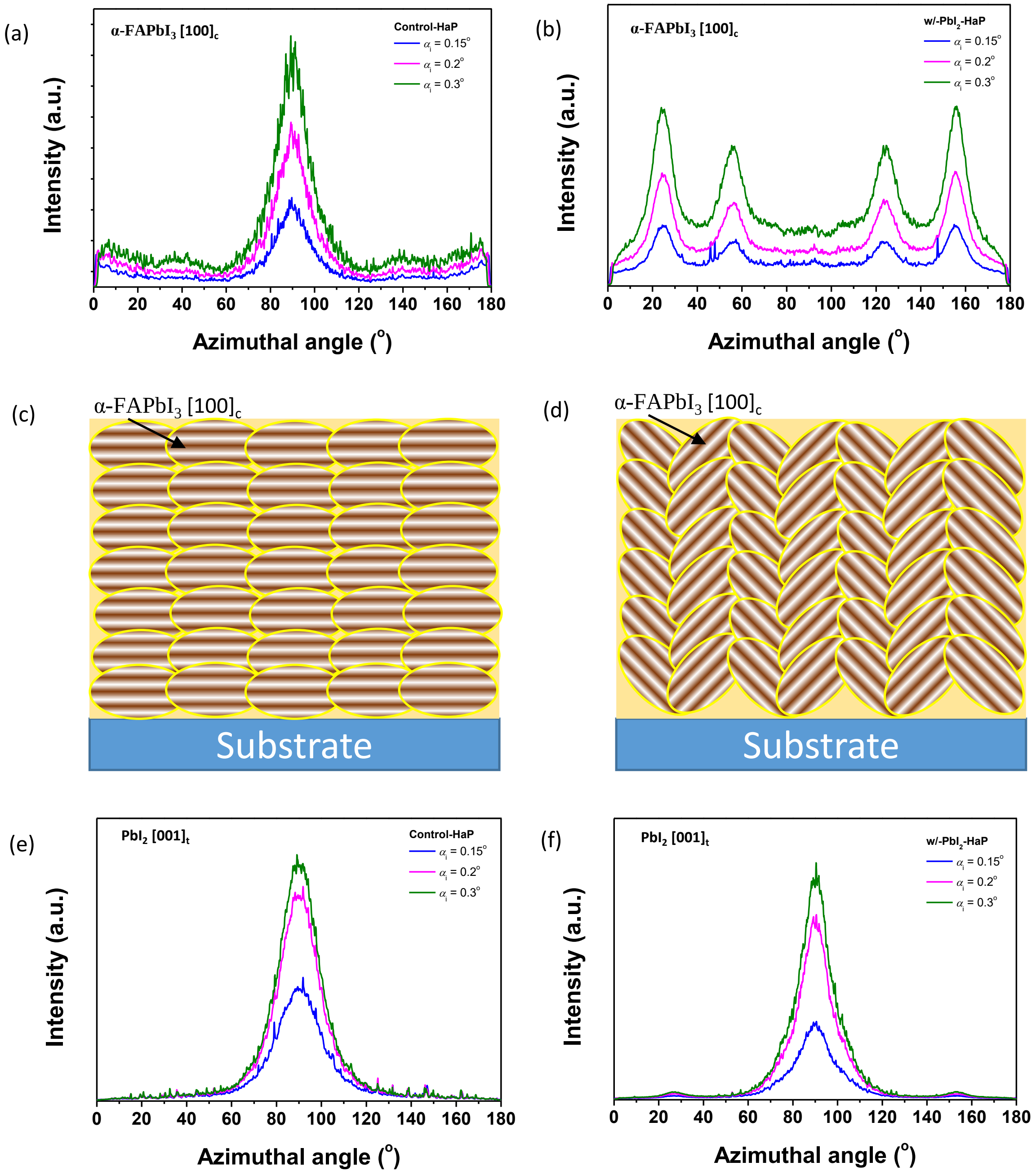

Supplementary Figure 8. (a and b) One-dimensional (1D) patterns of α-HaP [100]c at q_{xy} of 1.0/Å, (c and d) schemes of texturing of crystal domains for control- and w/-PbI_2-HaP, and (e and f) 1D patterns of PbI_2 $[001]_t$ at q_{xy} of 0.9/Å obtained from 2D-GIWAXS (Fig. 3).

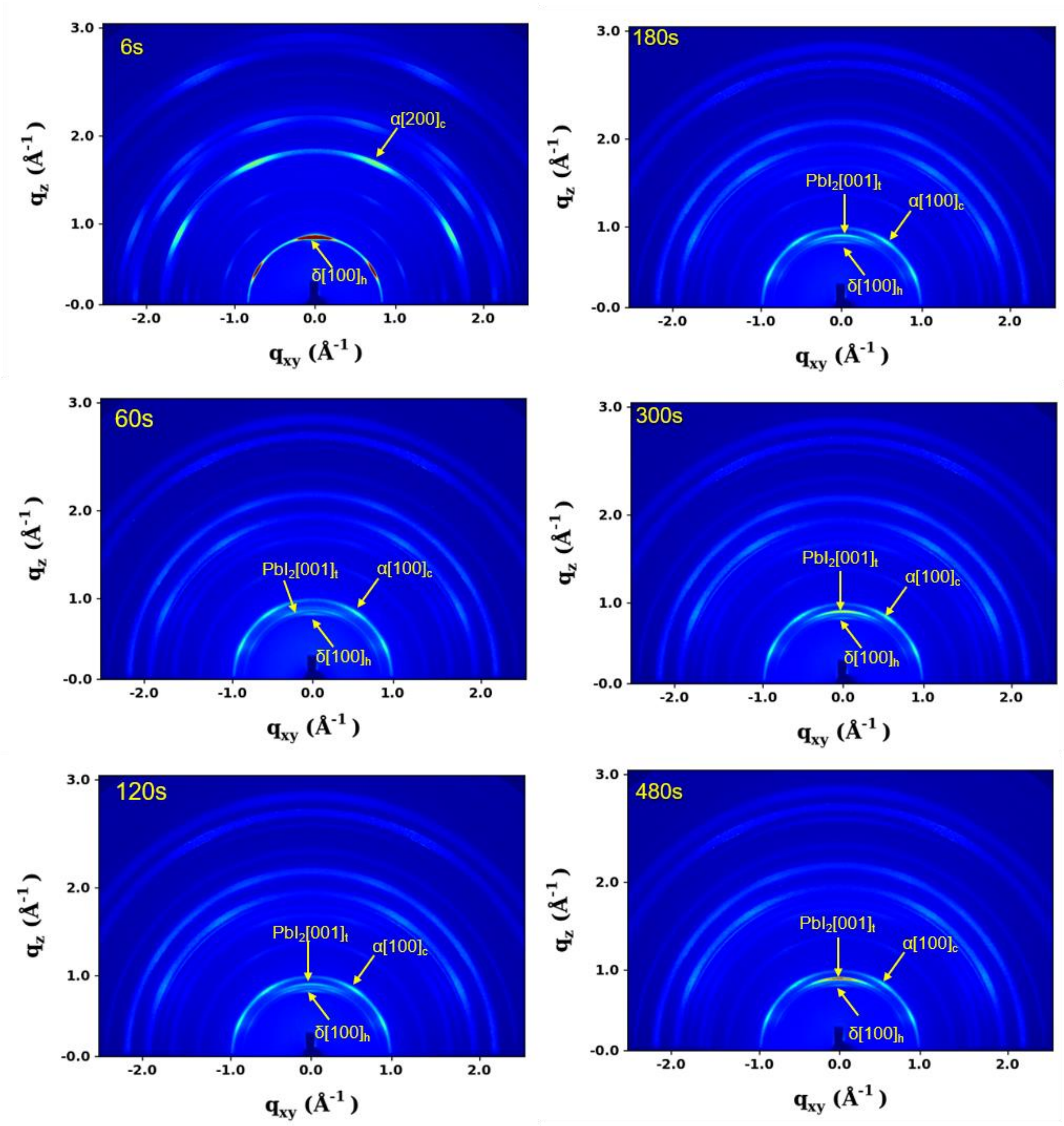

Supplementary Figure 9. 2D GIWAXS images of as-prepared w/PbI_2-HaP film observed over 6 s to 40 s with heating up to 150 °C.

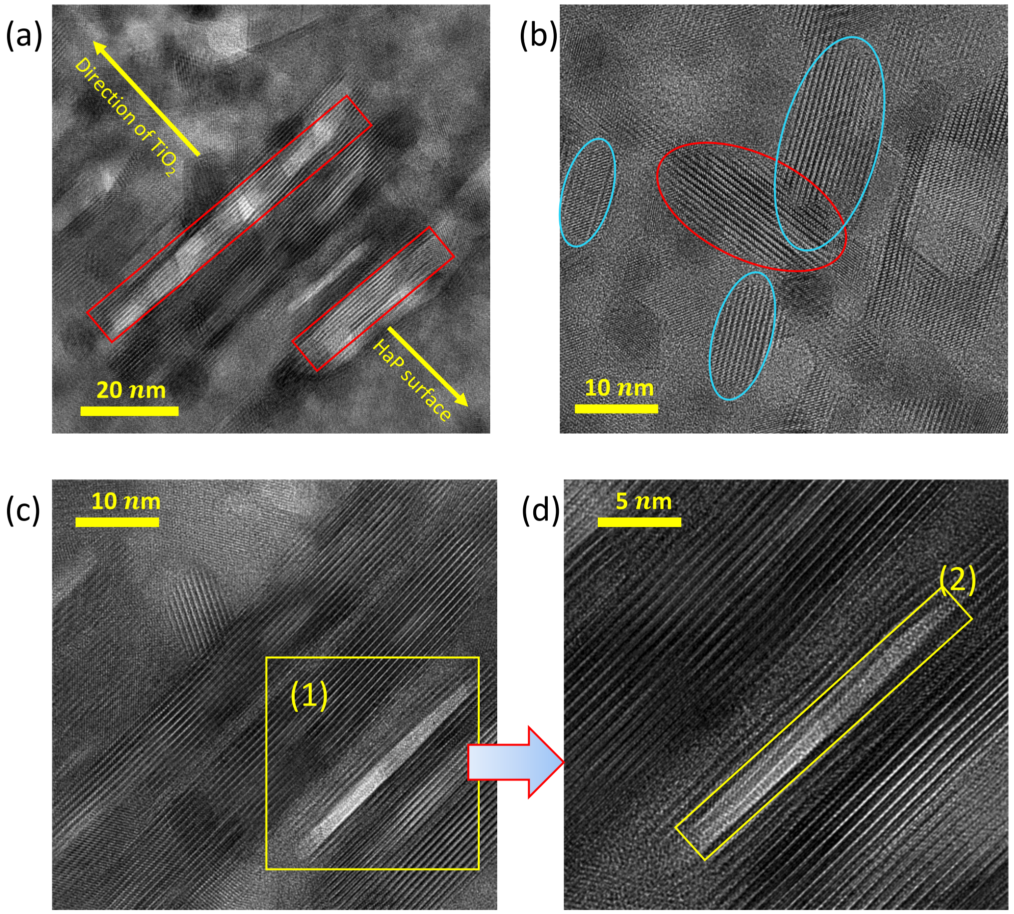

Supplementary Figure 10. HR-TEM images (a) PbI_2 located 200 nm deep from HaP film surface for control-HaP, (b) α-HaP crystal orientation (blue: out-of-plane, red: in-plane), and (c and d) magnified images for the remnant PbI_2.

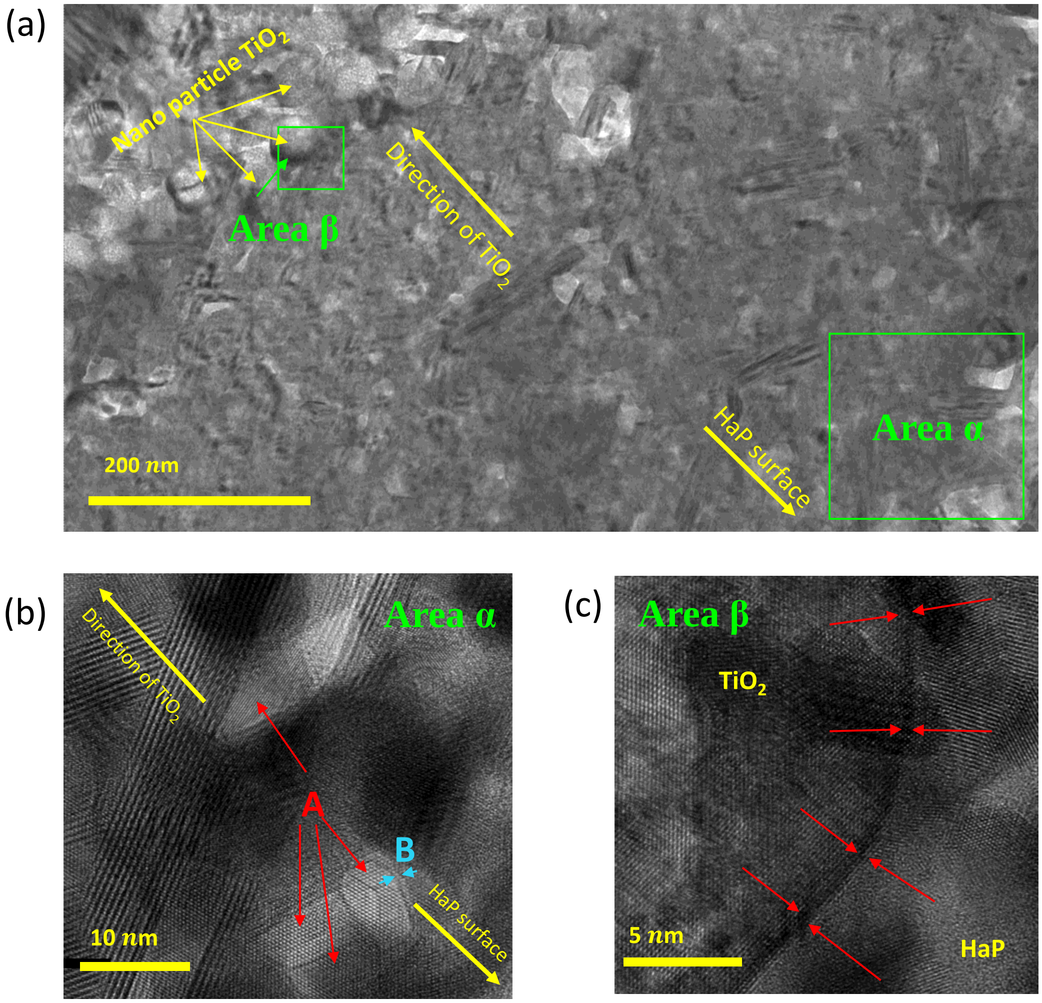

Supplementary Figure 11. HR-TEM images (a) low-magnified image for w/-PbI_2-HaP, (b) magnified image of area α for δ-HaP located between PbI_2 and α-HaP phase, (c) magnified image of area β for the observation of interface between TiO_2 and HaP in w/PbI_2 sample.

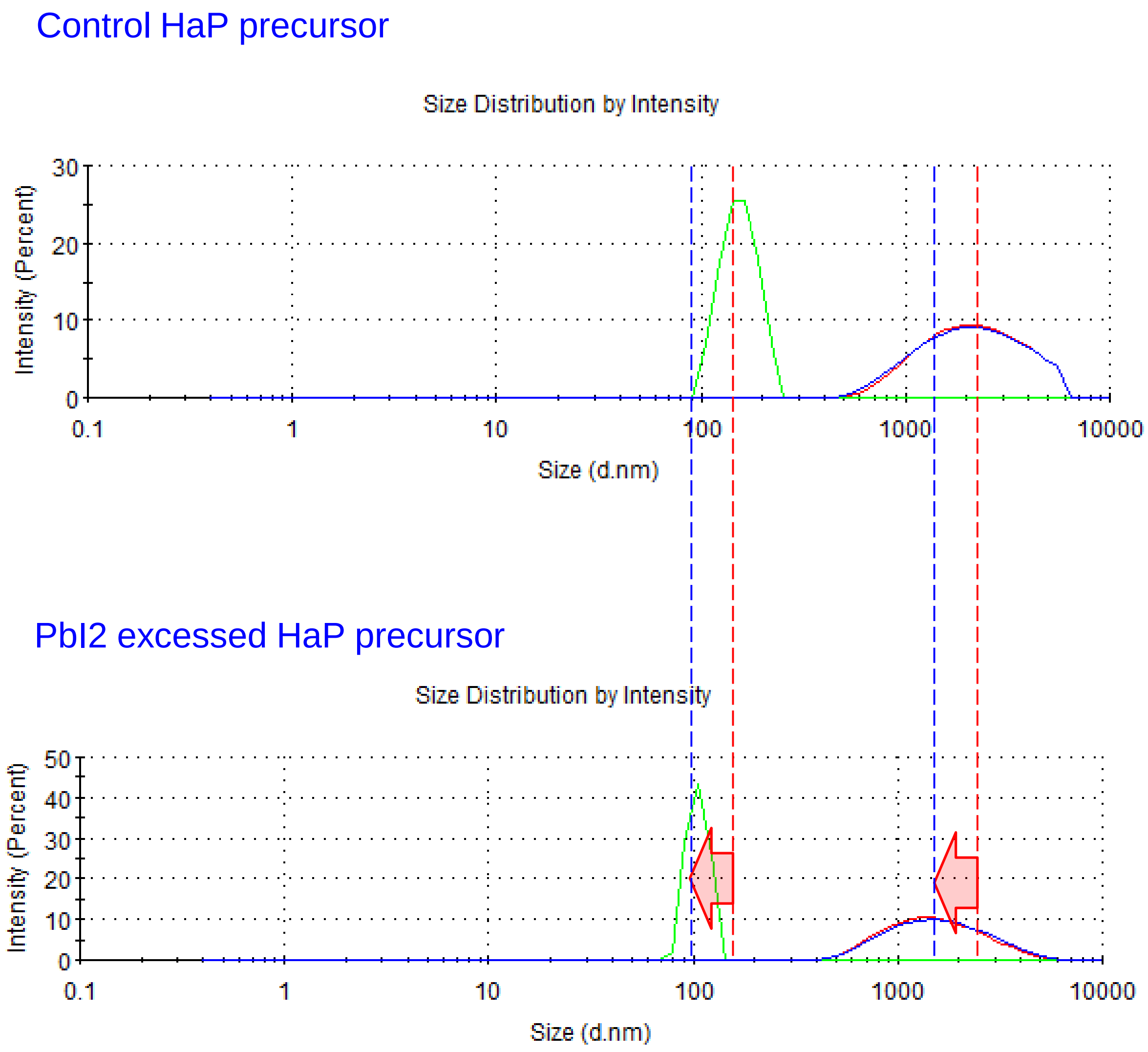

Supplementary Figure 12. Comparison of size and distribution of iodoplumbate complex measured by dynamic light scattering spectroscopy in control and w/PbI_2 precursor solution.

Supplementary References

(1) Liu, J., Saw, Robert E., Kiang, Y.-H., Calculation of Effective Penetration Depth in X-Ray Diffraction for Pharmaceutical Solids, *J. Pharm. Sci.* **99**, 3807–3814 (2010).
